# UBE2S promotes cell chemoresistance through PTEN-AKT signaling in hepatocellular carcinoma

**DOI:** 10.1038/s41420-021-00750-3

**Published:** 2021-11-16

**Authors:** Liang Gui, Sicai Zhang, Yongzi Xu, Hongwei Zhang, Ying Zhu, Lianbao Kong

**Affiliations:** 1 Department of General Surgery, Jiangsu Cancer Hospital & Jiangsu Institute of Cancer Research & The Affiliated Cancer Hospital of Nanjing Medical University, 210009 Nanjing, Jiangsu China; 2 Hepatobiliary Center, The First Affiliated Hospital of Nanjing Medical University, Key Laboratory of Liver Transplantation, Chinese Academy of Medical Sciences, 210029 Nanjing, Jiangsu Province China

**Keywords:** Cancer, Biomarkers

## Abstract

Ubiquitination displays a crucial role in various biological functions, such as protein degradation, signal transduction, and cellular homeostasis. Accumulating evidence has indicated that ubiquitination is essential in cancer progression. Ubiquitin-conjugating enzyme E2S (UBE2S) is a member of ubiquitin-conjugating enzyme family of the ubiquitin system and its role in hepatocellular cancer (HCC) is largely unknown. We investigated the role of UBE2S in HCC and found UBE2S upregulation is relevant with large tumor size, recurrence, and advanced TNM stage, serving as an independent risk factor of overall survival (OS) and disease-free survival (DFS) for HCC patients. We conducted in vitro experiments and found that in HCC cells, UBE2S overexpression increases the resistance to 5-FU and oxaliplatin, while UBE2S knockdown achieves an opposite effect. UBE2S is transcriptionally activated by the binding of FOXM1 to UBE2S promoter, which induces its upregulation and reduces PTEN protein level by promoting PTEN ubiquitination at Lys60 and Lys327 and facilitating AKT phosphorylation. The promotional effect of FOXM1-UBE2S axis on HCC cell chemoresistance is attenuated by allosteric AKT inhibitor, MK2206. In conclusion, our results reveal that UBE2S is a prognostic biomarker for HCC patients, and the FOXM1-UBE2S-PTEN-p-AKT signaling axis might be a promising target for the treatment of HCC.

## Introduction

Hepatocellular carcinoma (HCC) is a critical global health concern. Approximate 79 million new cases and 75 million HCC-related deaths annually occur worldwide [1, 2]. The prognosis of patients with HCC is poor, and tumor recurrence rate within five years after resection is over 70% [3, 4]. The combination of 5-fluorouracil (5-FU) and oxaliplatin is one of the commonly used chemotherapy regimens for HCC [5]. As one of the main risk factors, chemoresistance causes the failure of chemotherapy for HCC, accelerating tumor development and progression [6]. Therefore, it is imperative to explore the underlying mechanism involved in chemoresistance to develop better treatment strategy for HCC patients.

The ubiquitin-proteasome system (UPS) regulates a reversible biochemical process recruiting ubiquitin to degrade substrate proteins in cells [7]. Increasing evidence has demonstrated that UPS plays a vital role in cancer tumorigenesis and progression [8]. There are three types of enzymes in UPS including E1 ubiquitin-activating enzyme, E2 ubiquitin-conjugating enzyme, and E3 ubiquitin ligase and proteasomes, which are potential therapeutic targets for cancer and other diseases [9–11]. Ubiquitin-conjugating enzyme E2S (UBE2S), a member of E2 ubiquitin-conjugating enzyme which is also known as E2-EPF, elongates K11-linked polyubiquitin chain on APC/C substrates for 26S proteasome-mediated degradation to promote cell division [12–14]. UBE2S is also reported to be responsible for the ubiquitin-mediated proteasomal degradation of SOX2 to control the differentiation of mouse embryonic stem cells [15]. The crucial roles of UBE2S in cell proliferation, cell differentiation, and DNA repair inevitably implicate its involvement in tumor progression [16, 17]. Aberrant expression of UBE2S has been found in breast cancer [18], renal cell carcinoma [19], and cervical cancer [20]. In addition, UBE2S regulates β-Catenin through K11-linked polyubiquitination to mediate the malignant phenotypes in colorectal cancer [21]. However, biological functions of UBES2 in HCC remain largely unknown.

In this study, the expression and clinical significance of UBE2S were determined in TCGA-LIHC cohorts. The promotional role of UBE2S on the chemoresistance of HCC cells was revealed. Furthermore, our data indicate that UBE2S upregulation in HCC is induced by transcription factor FOXM1, and UBE2S renders PTEN ubiquitinated at Lys60 and Lys327, phosphorylating AKT to enhance HCC cell chemoresistance, which is attenuated by allosteric AKT inhibitor, MK2206. Collectively, we demonstrated an intriguing pathway that the activation of the FOXM1-UBE2S-PTEN-p-AKT signaling axis promotes HCC malignant phenotypes, suggesting that UBE2S may be a potential target for the treatment of HCC.

## Results

### UBE2S upregulation is positively related to poor prognosis of HCC patients

**Fig. 1:**
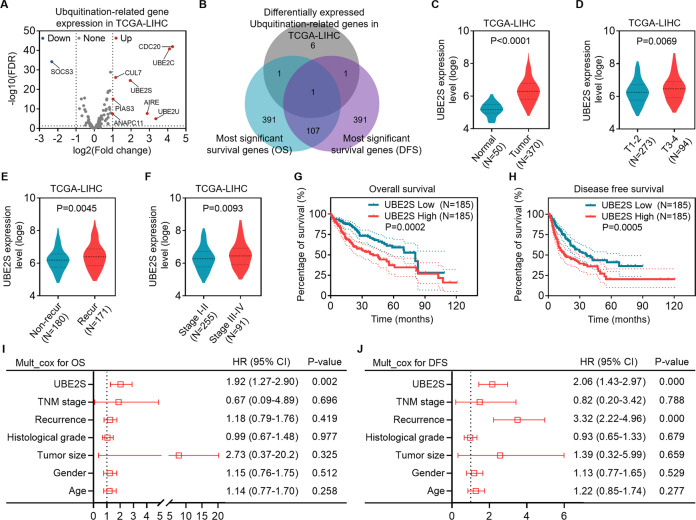
UBE2S is frequently upregulated in HCC, which is positively associated with a poor prognosis of patients with HCC. **A** Differentially expressed genes (DEGs) between HCC and non-tumoral tissues in TCGA-LIHC cohorts, involved in ubiquitination process, are shown in a volcano plot. **B** The overlap among ubiquitination-related DEGs, the genes most significantly related to overall survival (OS) and disease-free survival (DFS) in TCGA-LIHC database are displayed in a Venn diagram. **C** Difference in the level of UBE2S transcript between non-tumoral and HCC tissues is presented in a violin plot. **D**–**F** Difference in the level of UBE2S transcript between HCC subgroups, including tumor size (D, T1-2 vs. T3-4), recurrence event (E, non-recurrence vs. recurrence) and TNM stage (F, stage I–II vs. stage III-V) is presented in each violin plot, respectively. **G**, **H** The OS (**G**) and DFS (**H**) curves for patients with HCC are shown based on UBE2S transcript levels in HCC tissues in TCGA-LIHC cohorts. **I**, **J** The independent prognostic factor for OS (**I**) and DFS (**J**) of patients with HCC, based on multivariate Cox proportional hazard model, is showed in each forest plot, respectively.

To identify a novel ubiquitination-related driver gene for HCC, we analyzed the transcriptome profiling for HCC and non-tumoral liver tissues in TCGA database and screened for differentially expressed genes (DEGs) from KEGG gene sets involved in ubiquitin-mediated proteolysis (KEGG UBIQUITIN MEDIATED PROTEOLYSIS) in these tissues based on the criteria of false discovery rate (FDR) < 0.05 and fold change ≥2. We found that nine genes altered significantly in HCC compared with normal liver tissues (Fig. 1A). The most significant related genes of overall survival (OS) and disease-free survival (DFS) were screened by Gene Expression Profiling Interactive Analysis 2 (GEPIA2) website. Based on the overlap between the ubiquitination-related DEGs and the survival related genes, we finally found that the differential expression of UBE2S in HCC may be associated with the prognosis of patients with HCC (Fig. 1B). In addition to a higher expression level of UBE2S in HCC than the corresponding normal tissues (Fig. 1C), we confirmed that the transcript level of UBE2S was obviously higher in HCC with aggressive phenotypes, including large tumor size (Fig. 1D), recurrence (Fig. 1E), and advanced TNM stage (Fig. 1F). Kaplan–Meier survival analyses indicated that patients with high level of UBE2S trended to have lower OS and DFS rates (Fig. 1G, H). Moreover, multivariate Cox regression analyses revealed that high expression of UBE2S is an independent prognostic factor for poor OS and DFS of patients with HCC (Fig. 1I, J). Our data indicated that UBE2S is upregulated in HCC, which is correlated with malignant clinicopathological features and poor prognosis of patients with HCC.

### UBE2S induces chemotherapy resistance of HCC cells to 5-FU and oxaliplatin

**Fig. 2:**
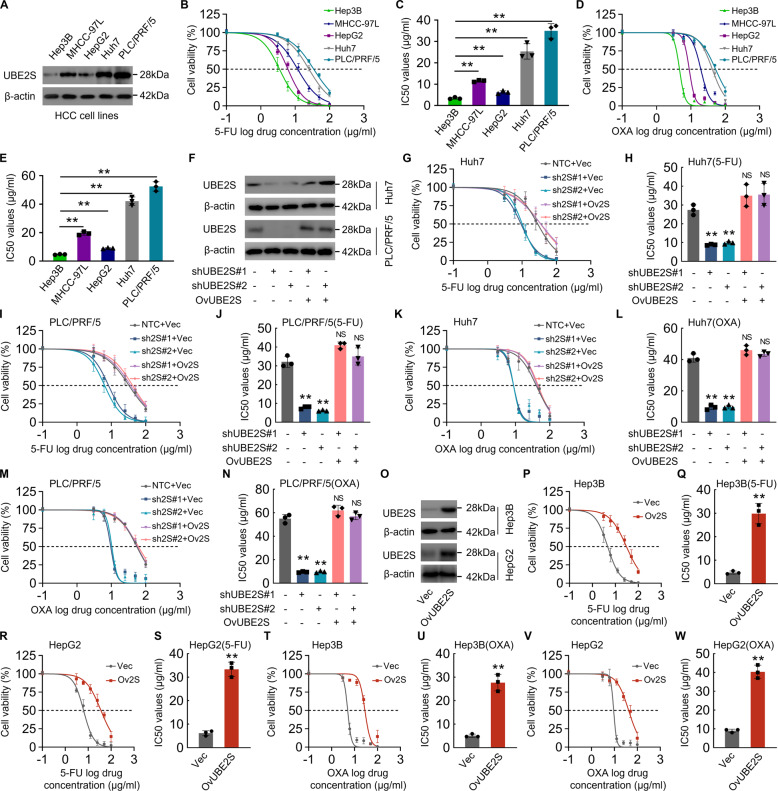
UBE2S enhances resistance of HCC cells to 5-FU and oxaliplatin. **A** UBE2S protein levels in five HCC cell lines were determined by western blot. **B**–**E** Cytotoxicity curves show the cytotoxic effects of 5-FU (**B**) and oxaliplatin (**D**) on five different HCC cell lines. Bar graphs (**C**, **E**) show the IC50 values of *n* = 3 independent experiments. **F** UBE2S knockdown and overexpression rescue were validated by western blot in Huh7 and PLC/PRF/5 cells, respectively. **G**–**J** Cytotoxic effects of 5-FU on UBE2S-knockdown and UBE2S-rescue (knockdown + overexpression) Huh7 (**G**, **H**) and PLC/PRF/5 (**I**, **J**) cells and control (NTC + Vec) cells were determined by CCK-8 assays, respectively. **K**–**N** Cytotoxic effects of oxaliplatin on UBE2S-knockdown and UBE2S-rescue Huh7 (**K**, **L**) and PLC/PRF/5 (**M**, **N**) cells and control cells were determined, respectively. **O** UBE2S overexpression efficiency was validated by western blot in Hep3B and HepG2 cells, respectively. **P**–**S** Cytotoxic effects of 5-FU on UBE2S-overexpression Hep3B (**P**, **Q**) and HepG2 (R and S) cells and vector control (Vec) cells were determined by CCK-8 assays, respectively. **T**–**W** Cytotoxic effects of oxaliplatin on UBE2S-overexpression Hep3B (**T**, **U**) and HepG2 (**V**, **W**) cells and Vec cells were determined, respectively.

To clarify the biological functions of UBE2S in HCC cells, the expression levels of UBE2S were determined in five HCC cell lines. The expression level of UBE2S was higher in PLC/PRF/5, Huh7 and MHCC-97L and lower in Hep3B and HepG2 cells (Fig. 2A). CCK-8 assays were used to examine the cytotoxic effects of 5-FU and oxaliplatin on these cell lines, showing that PLC/PRF/5, Huh7, and MHCC-97L cells were more resistant to oxaliplatin and 5-FU compared with Hep3B and HepG2 cells (Fig. 2B–E), indicating that the level of UBE2S is associated with the resistance of HCC cells to chemotherapy agents. Additionally, it also revealed that PLC/PRF/5, Huh7 and MHCC-97L cells had higher capabilities of migration and proliferation (Supplementary Fig. 1A–C), suggesting that UBE2S plays a critical role in regulating HCC development and progression. To further verify the correlation between UBE2S level and chemoresistance in HCC cells, we knocked down UBE2S in Huh7 and PLC/PRF/5 cells (Fig. 2F) and found that UBE2S knockdown suppressed the resistance of HCC cell to 5-FU and oxaliplatin (Fig. 2G–N). Also, it was showed that the suppression effect upon UBE2S knockdown on the resistance of HCC cells to 5-FU and oxaliplatin was rescued by the shRNA-resistant UBE2S overexpression (Fig. 2G–N). To further confirm the role of UBE2S in the chemoresistance of HCC cells, UBE2S was overexpressed in Hep3B and HepG2 cells (Fig. 2O). It showed that UBE2S-overexpressed HCC cells were more resistant to 5-FU and oxaliplatin (Fig. 2P–W). In conclusion, we deemed that UBE2S enhances the chemoresistance of HCC cells to chemotherapy drugs, 5-FU, and oxaliplatin.

### UBE2S is a critical effector of transcription factor FOXM1 in the regulation of HCC cell chemoresistance

**Fig. 3:**
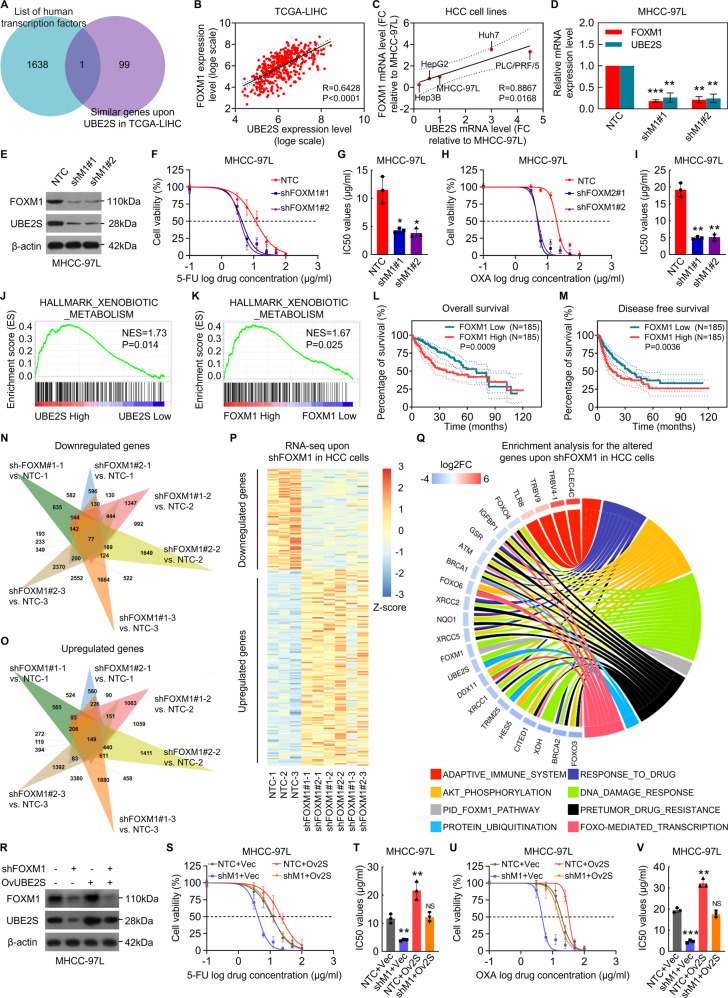
UBE2S is a critical effector for transcription factor FOXM1 in regulation of HCC cell chemoresistance. **A** The overlap between human transcription factors and UBE2S co-expressed genes in TCGA-LIHC database is displayed in a Venn diagram. **B**, **C** Analyses show linear regressions and Pearson correlations between UBE2S and FOXM1 transcript levels in HCC tissues in TCGA database (**B**) and in five different HCC cell lines (**C**). **D**, **E** UBE2S mRNA (**D**) and protein levels (**E**) were investigated in FOXM1-knockdown MHCC-97L cells. **F**–**I** Cytotoxic effects of 5-FU (**F**, **G**) and oxaliplatin (**H**, **I**) on FOXM1-knockdown MHCC-97L cells and corresponding NTC cells were determined, respectively. **J**, **K** Gene Set Enrichment Analysis **(**GSEA) plots show the enrichment of gene signatures involved in processing of cytotoxic agents and other xenobiotics between the HCC tissues with UBE2S^high^ and UBE2S^low^ (**J**), or the HCC tissues with FOXM1^high^ and FOXM1^low^ (**K**). **L**, **M** The OS (**L**) and DFS (**M**) curves for patients with HCC are shown based on FOXM1 transcript levels in HCC tissues in TCGA-LIHC cohorts. **N**, **O** Venn diagrams show significantly down- (**N**) and upregulated (**O**) DEGs (≥2 folds) in FOXM1 knockdown MHCC-97L cell as compared with NTC cells (*n* = 3). **P** A heat map of down- and upregulated DEGs between NTC and FOXM1-knockdown MHCC-97L cells (*n* = 3). **Q** A chord plot for the biological process enrichment involved by the down- and upregulated DEGs between NTC and FOXM1-knockdown MHCC-97L cells. **R**– **V** MHCC-97L cells were infected with the indicated lentiviral constructs, and then UBE2S and FOXM1 protein levels (**R**) and cytotoxic effects upon 5-FU (**S**, **T**) and oxaliplatin (**U**, **V**) were investigated.

The mechanisms of upregulation of UBE2S in HCC were further investigated. Firstly, we sought to find out whether the upregulation of UBE2S is induced by certain transcription factors. Based on the overlap between the list of human transcription factors and the co-expressed genes upon UBE2S according to TCGA-LIHC database, we found that transcription factor FOXM1 may positive correlated with UBE2S in HCC (Fig. 3A). To confirm the relationship between UBE2S and FOXM1 in HCC, the correlation of UBE2S with FOXM1 was analyzed in TCGA database and in HCC cell lines. As shown in Fig. 3B, C, UBE2S was significantly and positively correlated with FOXM1 in HCC tissues (*R* = 0.6428, *p* < 0.0001; Fig. 3B), as well as in HCC cell lines (*R* = 0.8867, *p* = 0.0168; Fig. 3C). To confirm the hypothesis, we investigated the mRNA and protein levels of UBE2S in FOXM1-knockdown HCC cells and found that the depletion of FOXM1 contributed to reduce mRNA and protein levels of UBE2S (Fig. 3D, E). In addition, we found that FOXM1 knockdown suppressed the resistance of HCC cell to 5-FU and oxaliplatin (Fig. 3F–I). The genes involved in processing of cytotoxic agents and other xenobiotics were enriched in high UBE2S expression group, which was similar with that in high FOXM1 expression group by performing the gene set enrichment analysis (GSEA) based on TCGA-LIHC database, which further supported that UBE2S is upregulated by FOXM1, promoting chemoresistance in HCC cells (Fig. 3J, K). Meanwhile, Kaplan–Meier survival analyses revealed that OS and DFS rates of HCC patients who have FOXM1^high^ tumor trend to be significantly lower (Fig. 3L, M). In addition, based on the RNA sequencing for FOXM1-knockdown HCC cells, we found that FOXM1 knockdown in HCC cells suppressed UBE2S mRNA level, FOXM1 pathway, AKT phosphorylation pathway, protein ubiquitination regulation as well as the drug response pathways (Fig. 3N–Q). To further investigate whether UBE2S is a key effector in the promotion of HCC cell chemoresistance regulated by FOXM1, we reconstituted FOXM1-knockdown MHCC-97L cells together with UBE2S overexpression. The ectopic expression of UBE2S antagonized the inhibition of HCC cell chemoresistance induced by FOXM1 knockdown (Fig. 3R–V). With all above, UBE2S is considered to be a critical effector of transcription factor FOXM1 in the regulation of resistance to cytotoxic agents in HCC cells.

### UBE2S transcription is activated by the binding of FOXM1 to UBE2S promoter

**Fig. 4:**
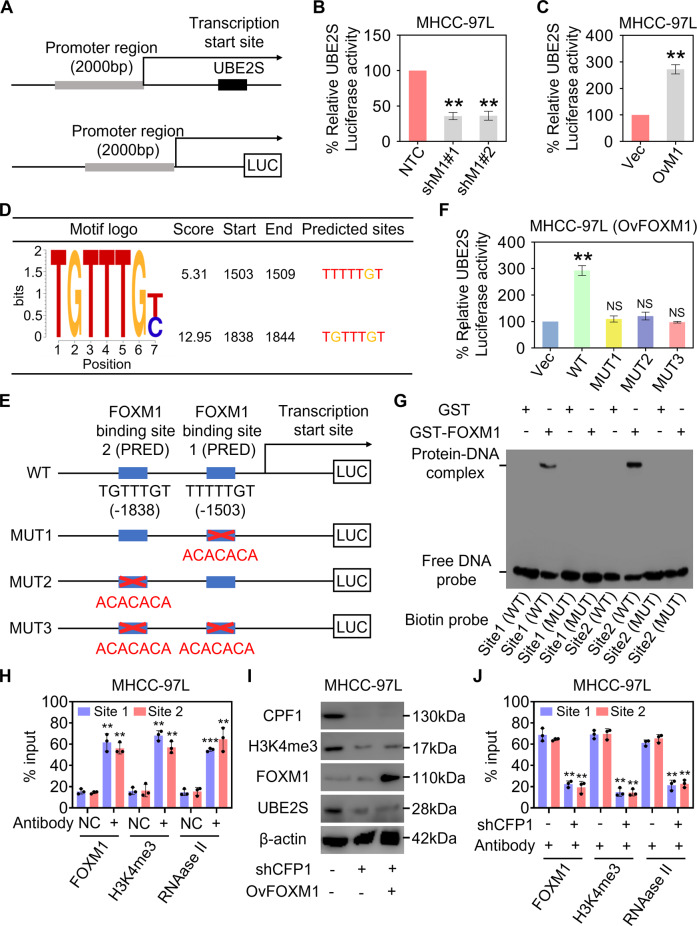
UBE2S transcription is activated by the binding of FOXM1 to UBE2S promoter. **A** Schematic representation of UBE2S promoter. **B**, **C** MHCC-97L cells stably silenced FOXM1 (**B**) or overexpressed FOXM1 (**C**) were transfected with indicated UBE2S wild-type reporter plasmids, and the luciferase activity was measured, respectively (*n* = 3). **D**, **E** Schematic representation of predicted FOXM1 binding sites (**D**) and corresponding mutated reporter constructs (**E**) for UBE2S. **F** FOXM1-overexpression MHCC-97L cells were transfected with indicated UBE2S wild-type (WT) or mutated reporter plasmids, and the luciferase activity was measured (*n* = 3). **G** Biotin-labeled DNA probe from the two predicted FOXM1 binding sites of UBE2S promoter region or corresponding mutant probe was incubated with GST-FOXM1 protein, and the DNA-protein complexes were separated on 6% native polyacrylamide gels. **H** ChIP-qPCR assays for FOXM1, H3K4me3, and RNA-polymerase II binding sites in the promoter of UBE2S gene (*n* = 3). **I** The protein levels of H3K4me3 and UBE2S were investigated in MHCC-97L cells stably silenced CPF1 together with FOXM1 overexpression. **J** ChIP-qPCR assays for FOXM1, H3K4me3, and RNA-polymerase II binding sites in the promoter of UBE2S gene in CPF1-knockdown MHCC-97L cells (*n* = 3).

Using the Eukaryotic Promoter Database (EPD) [22], a promoter region ~2000 bp upstream from the transcriptional start site of UBE2S gene was figured out (Fig. 4A). We constructed a luciferase gene reporter containing UBE2S promoter to explore whether the transcription of UBE2S is regulated by FOXM1 (Fig. 4A). As shown in Fig. 4B, C, FOXM1 knockdown decreased the activity of UBE2S promoter, while FOXM1 overexpression reversed this phenotype. Two potential binding sites for FOXM1 in the promoter region of UBE2S gene were predicted using JASPAR^2020^ program (Fig. 4D). Then, luciferase gene reporters containing the UBE2S promoter with FOXM1 binding site mutations were constructed (Fig. 4E). Mutations in the two binding sites for FOXM1 impeded the increase of UBE2S promoter activity mediated by FOXM1 overexpression (Fig. 4F). To further confirm the binding of these two sites with FOXM1, the recombinant glutathione S-transferase (GST)-FOXM1 fusion protein and biotin-labeled DNA probes for fragments of the UBE2S wild-type or mutated promoters were used for electrophoretic mobility shift (EMSA) assay. It was showed that the recombinant FOXM1 was able to bind the UBE2S wild-type promoter fragment and caused mobility shifts, and the binding was not found in the UBE2S mutated promoter fragment group (Fig. 4G). In addition, the mobility shift was not shown when the UBE2S wild-type or mutated promoter fragment was incubated with GST alone (Fig. 4G), implying that FOXM1 specifically binds to the promoter region of UBE2S that we predicted. Also, the binding of FOXM1 to the UBE2S promoter was confirmed by chromatin immunoprecipitation (ChIP) assays (Fig. 4H). RNA-polymerase II and H3K4 methylation and were used as marks for transcription activation [23, 24]. The binding of H3K4me3 and RNA-polymerase II to the UBE2S promoter were detected by ChIP assays (Fig. 4H). Although H3K4me3 is often known as an activating histone modification and assumed to play an instructive role in the transcription of genes, this field is still lacking a conserved mechanism to support this view. According to previous report, CFP1 acts an important role in histone H3-K4 methylation, and deletion of CFP1 in cells causes decreased H3K4me3 levels and transcription [25]. Then, the CFP1 was knocked down in HCC cells, and we checked the H3K4me3 levels. We found that CFP1 knockdown suppressed the H3K4me3 in HCC cells, as well as UBE2S levels (Fig. 4I). In addition, the suppression of UBE2S upon CFP1 knockdown cannot be rescued by FOXM1 overexpression (Fig. 4I). Furthermore, it was showed that the binding of FOXM1 and RNA-polymerase II to the UBE2S promoter were inhibited in the ChIP assay when there was a low level of H3K4me3 in HCC cells (Fig. 4J), reflecting that the transcription activation of UBE2S by FOXM1 is dependent on H3K4me3 activation. Taken together, these results show that UBE2S transcription is activated by the binding of FOXM1 to UBE2S promoter, regulating the downstream pathway.

### UBE2S ubiquitinates PTEN and FOXM1-UBE2S-PTEN-p-AKT pathway is required for HCC cell chemoresistance

**Fig. 5:**
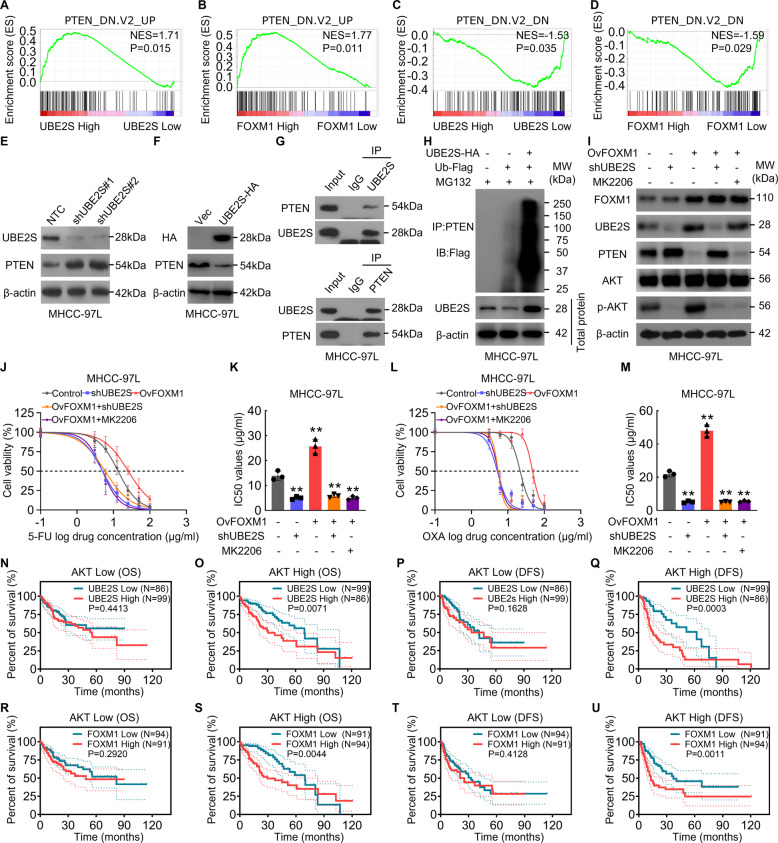
UBE2S promotes the ubiquitination of PTEN, enhancing HCC cell chemoresistance by activating AKT pathway. **A**, **B** GSEA plots show the enrichment of gene upregulated in cancer cells upon the knockdown of PTEN between the HCC tissues with UBE2S^high^ and UBE2S^low^ (**A**), or between the HCC tissues with FOXM1^high^ and FOXM1^low^ (**B**). **C**, **D** GSEA plots show the enrichment of gene downregulated in PTEN-knockdown cancer cells between the HCC tissues with UBE2S^high^ and UBE2S^low^ (**C**), or between the HCC tissues with FOXM1^high^ and FOXM1^low^ (**D**). **E**, **F** PTEN protein levels were investigated in UBE2S-knockdown (**E**) or UBE2S-overexpression (**F**) MHCC-97L cells. **G** UBE2S (Upper) and PTEN (Lower) complexes were co-immunoprecipitated with UBE2S and PTEN antibodies and immunoblotted with the indicated antibodies. **H** MHCC-97L cells overexpressed indicated proteins were treated with MG132 (10 μM) for 8 h, and the ubiquitination levels of PTEN protein were investigated. **I**–**M** MHCC-97L cells infected with indicated lentiviral plasmids and treated with MK2206 (5 μM) or DMSO (control) for 8 h. The indicated protein levels (**I**) and cytotoxic effects upon 5-FU (**J**, **K**) and oxaliplatin (**L**, **M**) were investigated. **N**, **O** The OS curves based on UBE2S transcript levels between HCC subgroup of AKT low (**N**) or high (**O**) expression. **P**, **Q** The DFS curves based on UBE2S transcript levels between HCC subgroup of AKT low (**P**) or high (**Q**) expression. **R**, **S** The OS curves based on FOXM1 transcript levels between HCC subgroup of AKT low (**N**) or high (**O**) expression. **T**, **U** The DFS curves based on FOXM1 transcript levels in HCC subgroup of AKT low (**T**) or high (**U**) expression.

It has been reported that UBE2S regulates cyclins as well as Wnt-β-Catenin pathway in some certain cancer cells [12, 21]. To further elucidate the mechanisms by which UBE2S upregulation modulates HCC cell chemoresistance, we conducted GSEA upon the expression levels of UBE2S in TCGA-LIHC database. However, we found that based on the GSEA results, the genes involved in cyclins as well as Wnt-β-Catenin pathway did not significantly enrich in UBE2S low or high HCC groups (Supplementary Fig. 2A–D). When retrospecting the RNA sequencing data in Fig. 3N–Q, it was emphasized that FOXM1 knockdown suppressed the AKT phosphorylation pathway rather than the AKT mRNA level. Additionally, in the GSEA results upon the TCGA RNA-seq data, we found that genes upregulated in cancer cells upon knockdown of PTEN were enriched in HCC tissues with high expression levels of UBE2S or FOXM1, while genes downregulated in PTEN-knockdown cancer cells were enriched in HCC tissues with low expression levels of UBE2S or FOXM1 (Fig. 5A–D). Taken together, these data indicated that the PTEN pathway may be a potential downstream responder upon the upregulation of UBE2S. Phosphatase and tensin homolog (PTEN) is reported to be a tumor suppressor gene acting through phosphating its downstream protein, such as AKT which involved in the regulation of malignant phenotypes including chemoresistance of cancer cells [26–28]. Hence, we raised the question whether the chemoresistance of HCC cells promoted by FOXM1-UBE2S is associated with PTEN. To ascertain the relationship between FOXM1/UBE2S and PTEN in HCC, we investigated the correlation between FOXM1/UBE2S and PTEN in the mRNA level in HCC cells, but no significant correlation was exhibited between FOXM1 and PTEN or between UBE2S and PTEN (Supplementary Fig. 2E, F). Then, the protein and mRNA levels of PTEN in UBE2S-knockdown HCC cells were investigated, suggesting that reduction of UBE2S increased protein levels of PTEN but did not alter the mRNA levels of PTEN (Fig. 5E and Supplementary Fig. 2G). Accordantly, overexpression of UBE2S reduced protein levels of PTEN in HCC cells (Fig. 5F). Furthermore, UBE2S has been reported to be implicated in PTEN interactome [29]. We then confirmed that PTEN was presented in UBE2S complexes in HCC cells (Fig. 5G). Interestingly, in vivo ubiquitination assay revealed that in the presence of MG132 (a specific proteasome inhibitor), overexpression of UBE2S increased the ubiquitination levels of PTEN (Fig. 5H). In addition, UBE2C and UBE2D both are reported to act as the priming E2 for UBE2S-mediated ubiquitination [30]. Based on this, it was assumed whether UBE2S cooperates with UBE2C and/or UBE2D to mediate ubiquitination of PTEN in HCC cells. However, our data showed that the PTEN protein levels were not altered in the UBE2C- or UBE2D-knockdown HCC cells (Supplementary Fig. 2H, I), suggesting that ubiquitination of PTEN mediated by UBE2S is independent from UBE2C and UBE2D in HCC.

Because PTEN antagonizes the AKT signaling pathway by dephosphorylating phosphoinositide [31, 32], we assessed levels of phosphorylation of AKT (p-AKT) in HCC cells treated by UBE2S-knockdown, FOXM1-overexpression and a combination of both. We found that the expression of p-AKT was decreased in UBE2S-knockdown HCC cells while increased in FOXM1-overexpression HCC cells (Fig. 5I). Also, upregulation of p-AKT upon FOXM1 overexpression in HCC cells was antagonized by UBE2S knockdown and an AKT inhibitor, MK2206 (Fig. 5I). Cell cytotoxicity assays showed that in UBE2S-knockdown and MK2206-treated HCC cells, FOXM1 overexpression failed to rescue the suppression effects on chemoresistance (Fig. 5J–M). These data indicate that UBE2S is specific and crucial for the PTEN-p-AKT signaling in chemoresistance. Furthermore, the Kaplan–Meier survival analyses revealed that, in patients who had high AKT expression, high level of UBE2S and FOXM1 correlates with lower OS and DFS rates. While in patients with low AKT expression, the level of UBE2S or FOXM1 did not show clinical significance in OS and DFS (Fig. 5N–U). In combination with our findings that FOXM1-UBE2S enhances cell chemoresistance through AKT signaling in HCC, these results demonstrate that the influence of UBE2S on the prognosis of patients with HCC is AKT-dependent. Therefore, a FOXM1-UBE2S-PTEN-p-AKT signaling axis enhancing HCC cell chemoresistance is revealed.

### UBE2S ubiquitinates PTEN at Lys60 and Lys327

**Fig. 6:**
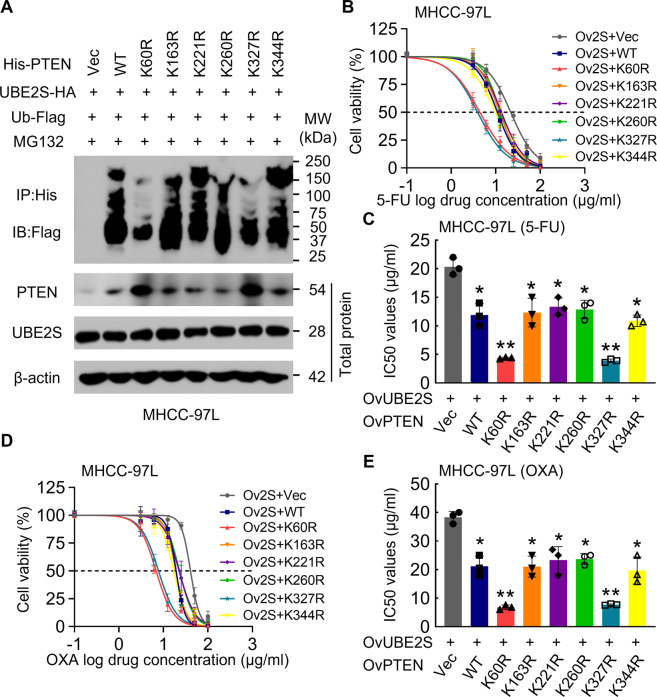
UBE2S ubiquitinates PTEN at Lys60 and Lys327. **A** MHCC-97L cells overexpressed indicated proteins were treated with MG132 (10 μM) for 8 h, and the ubiquitination levels of PTEN protein were investigated. **B**–**E** MHCC-97L cells infected with indicated lentiviral plasmids. The cytotoxic effects upon 5-FU (**B**, **C**) and oxaliplatin (**D**, **E**) were investigated (*n* = 3).

Since UBE2S-PTEN is the key link in FOXM1-UBE2S-PTEN-p-AKT signaling axis, we further investigated and identified the ubiquitination sites on the PTEN protein upon UBE2S. Based on the previous report [33], we figured out the potential ubiquitination sites (K60, K163, K221, K260, K327, and K344) on PTEN protein. We sought to determine whether these sites were targets of UBE2S-mediated PTEN ubiquitination. As demonstrated in the ubiquitination assay in Fig. 6A, the mutations of K60 and K327 to arginine acids (K60R and K327R) attenuated UBE2S-mediated ubiquitination (lanes 3 and 7), but the mutations of K163, K221, K260, and K344 did not affect UBE2S-mediated ubiquitination as compared with the wild-type control, suggesting that UBE2S-mediated PTEN ubiquitination occurs at Lys60 and Lys327. As the result in Fig. 5I–M showed that PTEN level was negative correlated with the HCC cell chemoresistance, we subjected the UBE2S overexpressed together with indicated PTEN wild-type and corresponding mutant overexpressed HCC cells to CCK-8 assays after 5-FU and oxaliplatin treatment. We found that the UBE2S overexpressed HCC cells were the most sensitive to 5-FU and oxaliplatin after overexpressing PTEN K60R and K327R mutant when compared to the other overexpression groups (Fig. 6B–E). Taken together, as the important part in FOXM1-UBE2S-PTEN-p-AKT axis, UBE2S-mediated PTEN ubiquitination occurs at Lys60 and Lys327, regulating the HCC cell chemoresistance.

## Discussion

In this work, we characterized that UBE2S is a critical prognostic factor for HCC patients. Although elevated UBE2S expression levels have been implicated in several cancers [18, 34, 35], the knowledge of the underlying mechanism of UBE2S upregulation in malignancies is limited. We found that in HCC, UBE2S is upregulated by FOXM1, which is a transcription factor of the Forkhead box (Fox) protein superfamily, participating in the oncogenesis of many cancers [36, 37]. Our results showed that UBE2S transcription is activated by the binding of FOXM1 to UBE2S promoter and dependent on H3K4me3 activation, which enhanced the resistance of HCC cells to 5-FU and oxaliplatin. The identification of FOXM1-UBE2S axis elucidates the activation of UBE2S in HCC.

Although the mechanism of UBE2S upregulation is clear, it is still unknown how UBE2S upregulation promotes the progression of HCC. Our results proved that compared with the negative control group, UBE2S-knockdown significantly increased the chemotherapy sensitivity of HCC cells to 5-FU and oxaliplatin, indicating that the upregulation of UBE2S enhances chemoresistance of HCC. Furthermore, the data from GSEA hinted that PTEN signaling is a potential pathway downstream of UBE2S. The pathways involving cancer chemoresistance are modulated by various positive and negative signals, which may be transduced primarily by PTEN inactivation [38–40]. Our study showed that UBE2S decreased the protein level of PTEN to enhance chemoresistance in HCC cells. We further found that UBE2S reduced the protein level of PTEN by promoting ubiquitination at Lys60 and Lys327. As known, PTEN-AKT is an important pathway regulating multiple biological processes such as drug resistance, metabolism, cell proliferation and DNA repair [41, 42]. We found that phosphorylation of AKT was decreased upon UBE2S knockdown while increased upon FOXM1 overexpression in HCC cells. In addition, upregulation of p-AKT and enhancement of HCC cell chemoresistance upon FOXM1 overexpression were antagonized by UBE2S knockdown and AKT inhibitor, indicating that UBE2S is crucial for the PTEN-p-AKT signaling in the regulation of HCC chemoresistance. Collectively, our findings reveal that UBE2S inhibited PTEN, leaded to the activation of AKT pathway, protected HCC cells from the cytotoxic effect of chemotherapy agents, implicating a novel role of UBE2S in the regulation of cell chemoresistance.

For the clinical importance in this study, we found that the UBE2S was an independent predictor for OS and DFS of patients with HCC. These findings underscored a potentially crucial role of UBE2S in the poor prognosis of patients with HCC. More excitingly, we found that when grouping patients by the levels of AKT transcript in HCC tissues, in patients with low AKT level, high levels of UBE2S no longer show the clinical significance in poor prognosis. The clinical significance of FOXM1-UBE2S-PTEN-p-AKT axis in HCC has been identified in our study.

In summary, we found that UBE2S acts as a biomarker of the prognosis for patients with HCC. The role of UBE2S in the chemoresistance of HCC is elucidated preliminarily, for the upregulation of UBE2S promotes the resistance of HCC cells to 5-FU and oxaliplatin through FOXM1-UBE2S-PTEN-p-AKT axis. The FOXM1-UBE2S-PTEN-p-AKT pathway might be a target for HCC treatment.

## Materials and methods

### Cell lines and cell culture

HCC cell lines (Hep3B, MHCC-97L, HepG2, PLC/PRF/5, and Huh7) were purchased from the Cell Resource Center, Chinese Academy of Science Committee (Shanghai, China). These cells were cultured in Dulbecco’s Modified Eagle’s Medium (DMEM, Gibco, USA), supplemented with 10% fetal bovine serum (FBS, Hyclone, USA) and 1% penicillin-streptomycin cocktail (Invitrogen, USA) with 5% CO_2_ in a humidified 37 °C incubator. For the stable knockdown and overexpression of corresponding genes in HCC cell lines, specific vectors were transfected into cells using lentiviral infection system, and the transfected cells were selected with puromycin (3 μg/ml) or blasticidin (5 μg/ml).

### Constructs

Specific shRNAs targeting UBE2S or FOXM1 were cloned into pLKO.1-puro vector (Addgene, USA) for stable knockdown, and the sequences including negative control (NTC) were listed in the supplementary material 1. Full-length coding sequence (CDS) for UBE2S, FOXM1, and Ub protein were cloned into pLenti-CMV-blast vectors (Addgene, USA) for stable overexpression, and the primers for amplification for full-length CDS of these genes were listed in the supplementary material 1. For luciferase assay, wild-type (WT) and mutated (MUT) promoter region ~2000 bp upstream of the transcriptional start site of UBE2S gene were cloned into the pGL3-Basic luciferase reporter vectors (Promega, USA), and the primers were listed in the supplementary material 1.

### Western blotting

HCC cell lysates were separated by 10% sodium dodecyl sulfate-polyacrylamide gel electrophoresis (SDS-PAGE), electroblotted onto polyvinylidene fluoride (PVDF) membranes, incubated with indicated specific primary antibodies overnight at 4 °C, and then incubated with corresponding secondary antibodies at room temperature. The primary antibodies included UBE2S (1:1000, ab177508, abcam, UK), FOXM1 (1:1000, ab264210, abcam, UK), CPF1 (1:1000, PA5-112012, Invitrogen, USA), H3K4me3 (1:1000, ab8580, abcam, UK), HA (1:2000, 26183, Invitrogen, USA), FLAG (1:2000, ab205606, abcam, UK), PTEN (1:1000, ab267787, abcam, UK), UBE2C (1:1000, ab252940, abcam, UK), UBE2D (1:1000, ab249927, abcam, UK), AKT (1:1000, ab8805, abcam, UK), phosphor-AKT (phospho S473, 1:1000, ab81283, abcam, UK), and β-actin (1:5000, sc-81178, Santa Cruz, USA).

### CCK‐8 assay

The CCK-8 assay was conducted as the method described previously [43]. HCC cells were incubated with 5-FU or oxaliplatin in 96-well plate for 48 h, and the culture medium was replaced with 100 μL of fresh culture medium containing 10 μL of CCK‐8 regent (Beyotime Biotechnology, China). The plate was incubated in an incubator for 2 h, then the optical density (OD) was measured with microplate reader (Bio‐Rad, USA) at a wavelength of 450 nm. The procedures were repeated for three times. Cell survival rates were calculated. Probit regression analysis was used to calculate Half maximal inhibitory concentration (IC50) by GraphPad Prism.

### Quantitative real-time PCR

Complementary DNA was synthesized from total RNA using the PrimeScript RT reagent Kit (Takara, Japan). SYBR Premix ExTaq (Takara, Japan) was used to perform quantitative real-time PCR. GAPDH was used as an internal reference for calculating the relative expression levels of indicated genes. For each gene, the average of three independent analyses was calculated. The corresponding primers were listed in the supplementary material 1.

### Luciferase assay

Constructs of UBE2S firefly luciferase reporter were transfected into cells with stable knockdown or overexpression of corresponding genes for 48 h. Using the pBIND vector acts as the internal control (Promega, USA); Renilla luciferase activity was used to normalize the activity of firefly luciferase, which was measured for at least three times by the dual-luciferase reporter assay system (Promega, USA).

### RNA sequencing

Using the High Pure RNA isolation kit (Invitrogen, USA), RNA was extracted form indicated HCC cells with three independent replicates. According to the manufacturer’s protocol, whole transcriptome libraries were prepared with the TruSeq Stranded Total RNA Sample Preparation kit (Illumina, USA). Briefly, 500 ng total RNA was removed rRNA using the Ribo-Zero Gold kit (Illumina, USA) and then it was purified for end repair and 5’-adapter ligation. After that, it was performed by reverse transcription with random primers containing randomized hexamers as well as 3′-adapter sequences. In the end, the cDNAs were purified and amplified. The libraries were subjected for sequencing on Illumina High HiSeq 2500 with paired-end 50 base pair long reads. Alignment was performed by HISAT2 [44]. The HTSeq [45] was used to compare transcript levels between NTC and FOXM1-knockdown groups based on FPKM with log2(fold change) ≥1 or ≤−1 and *P* < 0.05. The RNA sequencing data were listed in supplementary material 2.

### Chromatin immunoprecipitation-qPCR assay

We used an EZ-ChIP Assay Kit (Upstate, USA) to perform the ChIP assay according to the instructions of the manufacturer and previous reports [46] with minor modifications. Protein-DNA complexes crosslinked by formaldehyde were sheared with ultrasound pulses on wet ice, then precipitated with anti-UBE2S (ab177508, abcam, UK), control IgG (ab37415, abcam, UK), H3K4me3 (ab8580, abcam, UK), and RNA polymerase II CTD repeat YSPTSPS (ab5131, abcam, UK) antibodies repectively. Specific primer sets for UBE2S, H3K4me3, and RNA polymerase II CTD repeat YSPTSPS binding sites in the promoter region of the UBE2S gene were used to perform qPCR.

### Protein expression and electrophoretic mobility shift assay

Recombinant human GST-FOXM1 (GST, N-Terminal) protein was purchased from Novus Biologicals (USA). The fragments of ~60 bp containing putative FOXM1 binding sites in the promoter of UBE2S were synthesized and labeled with biotin at the 5′ end from Beyotime Biotechnology (China). Using a LightShift Chemiluminescent EMSA kit (Invitrogen, USA), EMSA assay was performed as previously described [47]. In brief, the binding reaction mixture (20 μL) contained 50 ng of purified protein, 1 pmol of labeled probe, 1 mM DTT, 100 mM KCl, 0.1 mM ethylenediaminetetraacetic acid (EDTA), 25 mM HEPES-KOH (pH = 7.5), 20% glycerol, and 4 mg of poly (dI-dC) to minimize nonspecific interactions. The assay mixtures were incubated at room temperature for 30 min. ChemiDoc™ MP Imaging System (Bio-Rad, USA) was used to detect Biotin-labeled DNA. The sequences of fragments which were used in the EMSA assay were listed in the supplementary information.

### Coimmunoprecipitation

HCC cells were lysed by NETN buffer (pH 8.0) which contains 0.5% Nonidet P-40, 100 mM NaCl, 1 mM EDTA, 20 mM Tris-HCl, 10 mM NaF, 50 mM b-glycerophosphate, and 1 mg/mL of pepstatin A and aprotinin each. After centrifugation at 12,000 rpm for 10 min, cell lysates were incubated with indicated antibody and protein A/G PLUS agarose beads (Santa Cruz) overnight or at 4 °C for 4 h. The immunocomplexes were washed with NETN buffer after incubation, then separated by SDS-PAGE.

### In vivo ubiquitination assay

The in vivo ubiquitination assay was performed according to the procedures previously described [11]. The indicated gene co-overexpression cells were treated with 10 μM MG132 for 8 h before harvest. The cells were lysed using NETN buffer described above. PTEN was immunoprecipitated using PTEN antibody (ab267787, abcam, UK) or His antibody (MA1-135, Invitrogen, USA) as well as protein A/G agarose beads (Santa Cruz, USA). Ubiquitinated PTEN was detected using anti-Flag antibody in the western blot.

### Bioinformatics analysis

The data of RNA sequencing (level 3) were obtained from the Cancer Genome Atlas Database (TCGA, https://portal.gdc.cancer.gov/). There were 370 tumor and 50 non-tumoral samples in TCGA-LIHC dataset. The genes involved in ubiquitin-mediated proteolysis were downloaded from the gene set of KEGG UBIQUITIN MEDIATED PROTEOLYSIS. The top 500 genes potentially related to the overall survival (OS) and disease-free survival (DFS) of HCC patients were analyzed from the GEPIA2 online database [48]. The functional enrichment analyses upon the levels of UBE2S or FOXM1 in HCC were conducted by the Gene Set Enrichment Analysis (GSEA). The online tool jvenn was used to generate the Venn diagram [49]. Using the OmicStudio tools, the heat map analysis was performed at https://www.omicstudio.cn/tool. The promoter region of UBE2S were analyzed by the Eukaryotic Promoter Database (EPD) [22]. Potential binding sites for transcription factor FOXM1 in the promoter region of UBE2S gene were predicted by the JASPAR^2020^ program (http://jaspar.genereg.net).

### Statistical analysis

The data analysis was conducted with Statistical Program for Social Sciences 20.0 software (SPSS, USA) and GraphPad Prism 8.0 (GraphPad Software, USA). The correlation of indicated genes was examined with Pearson’s correlation test. Comparisons between groups were did with two-tailed independent Student’s *t*-test and Mann–Whitney *U*-test. Survival rates were analyzed by Kaplan–Meier plot and log-rank test. The data were presented as mean ± SD unless stated otherwise. Statistically significance was considered to be reached at *p < 0.05, **p < 0.01, ***p < 0.001.

## Supplementary information

Supplemental Figure 1

Supplemetary Figure2

Supplementary material 1

Supplementary material 2

declaration

## Data Availability

The data in this work are available with the approval of corresponding authors in reasonable requirement.
