# Supplementary figures and images for: UBE2S promotes cell chemoresistance through PTEN-AKT signaling in hepatocellular carcinoma
Source: Cell Death Discov. 2021 Nov 16;7:357. doi: 10.1038/s41420-021-00750-3 (PMC8595659; doi:10.1038/s41420-021-00750-3)

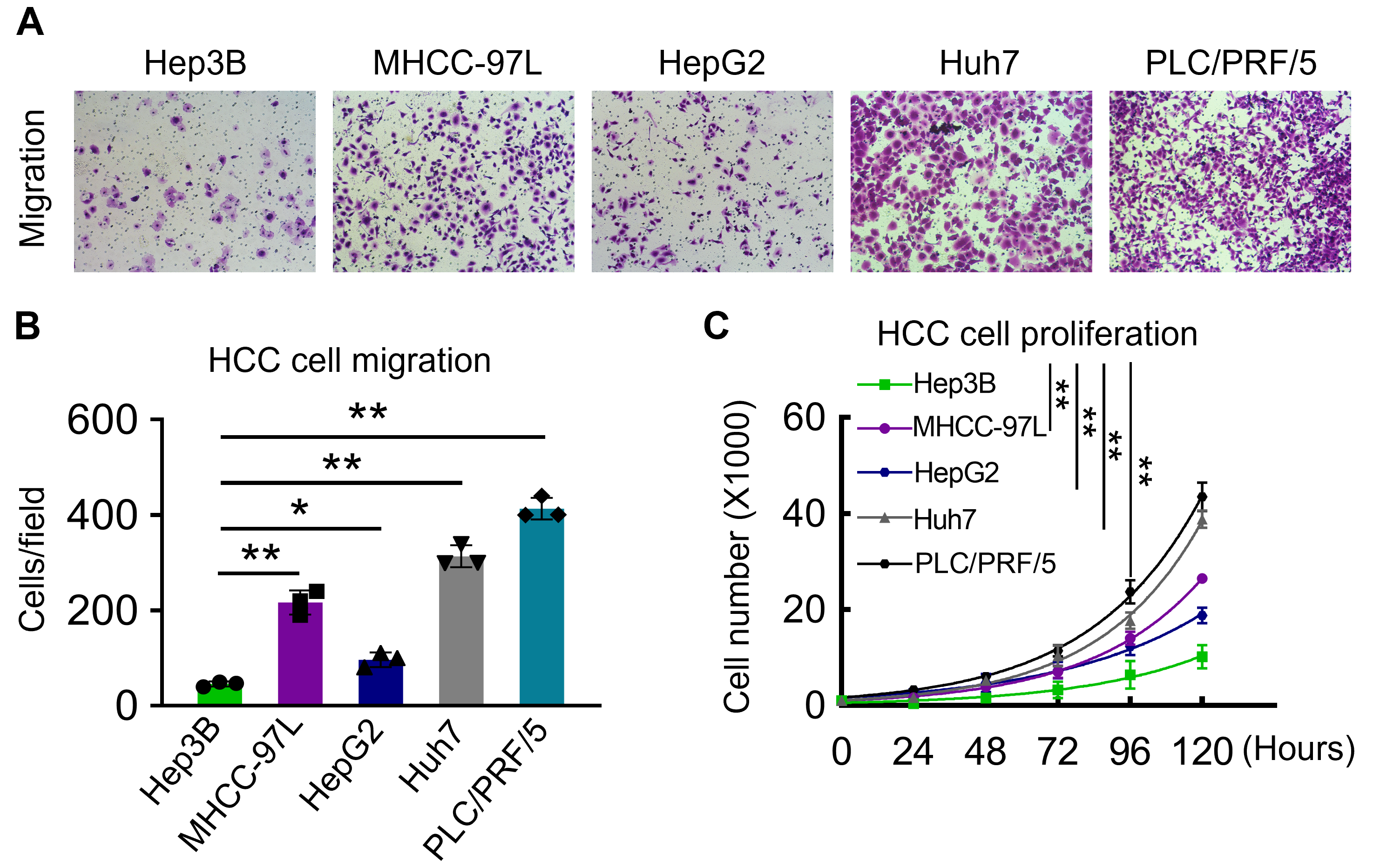

Supplement: Supplementary file 1 — Supplemental Figure 1 [file 41420_2021_750_MOESM1_ESM.tif]

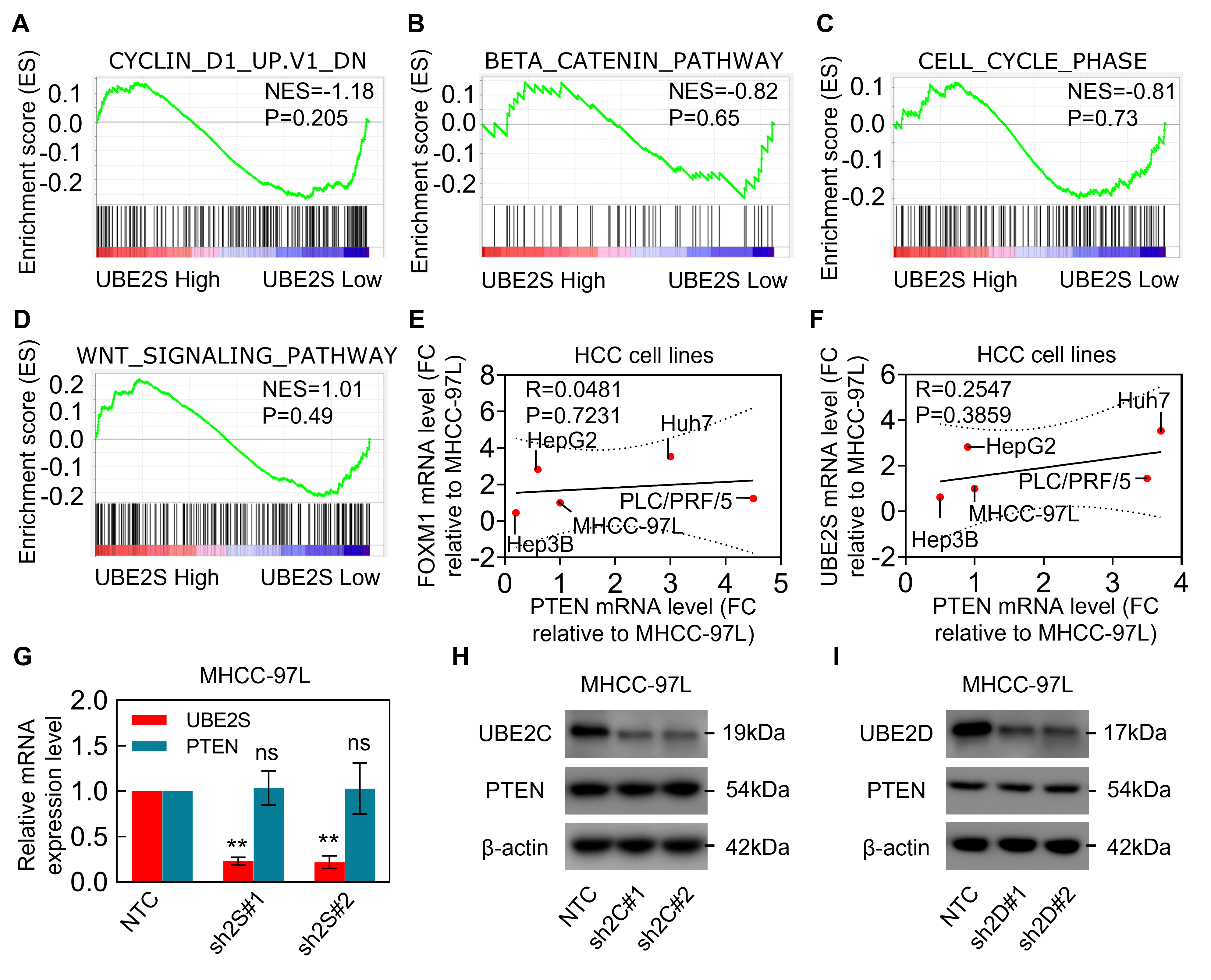

Supplement: Supplementary file 2 — Supplemetary Figure2 [file 41420_2021_750_MOESM2_ESM.tif]
