# Supplementary material for: UBE2S promotes cell chemoresistance through PTEN-AKT signaling in hepatocellular carcinoma
Source: Cell Death Discov. 2021 Nov 16;7:357. doi: 10.1038/s41420-021-00750-3 (PMC8595659; doi:10.1038/s41420-021-00750-3)
Supplement: Supplementary file 3 — Supplementary material 1 [file 41420_2021_750_MOESM3_ESM.docx]

**shRNA sequences**

shNTC:

Sense: 5’-CCGGAAGCTGACCCTGAAGTTCATTTCAAGAGAATGAACTTCAG

GGTCAGCTTTTTTTT-3’

Anti-sense: 5’-AATTAAAAAAAAGCTGACCCTGAAGTTCATTCTCTTGAAATG

AACTTCAGGGTCAGCTT-3’

shUBE2S#1:

Sense: 5’-CCGGGCAGCAGTGGGTCCATCTAGCTTCAAGAGAGCTAGATGGA

CCCACTGCTGCTTTTTT-3’

Anti-sense: 5’-AATTAAAAAAGCAGCAGTGGGTCCATCTAGCTCTCTTGAAGC

TAGATGGACCCACTGCTGC-3’

shUBE2S#2:

Sense: 5’-CCGGGGTGCCAGCAGTCTGGCATGATTCAAGAGATCATGCCAGAC

TGCTGGCACCTTTTTT-3’

Anti-sense: 5’-AATTAAAAAAGGTGCCAGCAGTCTGGCATGATCTCTTGAATC

ATGCCAGACTGCTGGCACC-3’

shFOXM1#1:

Sense: 5’-CCGGGCTCCGCCGGCGCCAATTTCATTCAAGAGATGAAATTGGCG

CCGGCGGAGCTTTTTT-3’

Anti-sense: 5’-AATTAAAAAAGCTCCGCCGGCGCCAATTTCATCTCTTGAATG

AAATTGGCGCCGGCGGAGC-3’

shFOXM1#2:

Sense: 5’-CCGGGCCGGCGCCAATTTCAAACAGTTCAAGAGACTGTTTGAAAT

TGGCGCCGGCTTTTTT-3’

Anti-sense: 5’-AATTAAAAAAGCCGGCGCCAATTTCAAACAGTCTCTTGAACT

GTTTGAAATTGGCGCCGGC-3’

shUBE2C#1:

Sense: 5’- CCGGGTCTGGCGATAAAGGGATTTCTTCAAGAGAGAAATCCCTTT

ATCGCCAGACTTTTTT-3’

Anti-sense: 5’- AATTAAAAAAGTCTGGCGATAAAGGGATTTCTCTCTTGAAGA

AATCCCTTTATCGCCAGAC-3’

shUBE2C#2:

Sense: 5’- CCGGGCAGCTGGAACAGTATATGAATTCAAGAGATTCATATACTG

TTCCAGCTGCTTTTTT-3’

Anti-sense: 5’- AATTAAAAAAGCAGCTGGAACAGTATATGAATCTCTTGAATT

CATATACTGTTCCAGCTGC-3’

shUBE2D#1:

Sense: 5’-C CCGGGCAACTGCATAACAGCCTTCCTTCAAGAGAGGAAGGCTG

TTATGCAGTTGCTTTTTT-3’

Anti-sense: 5’- AATTAAAAAAGCAACTGCATAACAGCCTTCCTCTCTTGAAGG

AAGGCTGTTATGCAGTTGC-3’

shUBE2D#2:

Sense: 5’- CCGGGGCGAATCCAGAAGGAGTTAGTTTCAAGAGAACTAACTCC

TTCTGGATTCGCTTTTTT-3’

Anti-sense: 5’- AATTAAAAAAGCGAATCCAGAAGGAGTTAGTTCTCTTGAAA

CTAACTCCTTCTGGATTCGC-3’

**Vector construction primers**

pLenti-CMV-blast-UBE2S-HA:

Forward: 5’-GGGGATCCatgaactccaacgtggagaac-3’

Reverse: 5’-GG ctcgagTTAAGCGTAATCTGGAACATCGTATGGGTACAGCC

GCCGCAGCGCCCGCTTCTT-3’

pLenti-CMV-blast-Ub-Flag:

Forward: 5’-GGGGATCCGCCACCATGCAGATCTTCGTGAAAACCC-3’

Reverse: 5’-GGctcgagTTACTTGTCATCGTCGTCCTTGTAGTCGCCACCCCT

CAGGCGCAGG-3’

pLenti-CMV-blast-FOXM1:

Forward: 5’- GGGGATCCGCCACCATGAAAACTAGCCCCCGTCGGCCA-3’

Reverse: 5’- GGctcgagAGTTTATTCCTGAGCTACAGTAG-3’

pGL3-Basic-UBE2S-promoter (WT)-reporter:

Forward: 5’- ctcgagGGACGTGATGAGATTCAGTAGATGA-3’

Reverse: 5’- aagcttCGGCCGCGCGCGCACCACTGCCTCT-3’

pGL3-Basic-UBE2S-promoter (MUT1)-reporter:

Forward: 5’- AACACACCTGGCTAAACACACAATTTTTAGTACAGAT-3’

Reverse: 5’- ATCTGTACTAAAAATTGTGTGTTTAGCCAGGTGTGTT-3’

pGL3-Basic-UBE2S-promoter (MUT2)-reporter:

Forward: 5’- CAGTTGTTTTGGGTTACACACATTTGTACATATTGGT-3’

Reverse: 5’- ACCAATATGTACAAATGTGTGTAACCCAAAACAACTG-3’

**qPCR primers**

UBE2S:

Forward: 5’-GACCAAGATCTTCCACCCGA-3’

Reverse: 5’- GCCGCATACTCCTCGTAGTT-3’

FOXM1:

Forward: 5’-AGTTCTGATGGACTGGGCTC-3’

Reverse: 5’-CTCTCAGTGCTGTTGATGGC-3’

PTEN:

Forward: 5’-TGGATTCGACTTAGACTTGACCT-3’

Reverse: 5’-GGTGGGTTATGGTCTTCAAAAGG-3’

GAPDH:

Forward: 5’-AGAAGGCTGGGGCTCATTTG-3’

Reverse: 5’-AGGGGCCATCCACAGTCTTC-3’

**ChIP-qPCR primers**

Binding site 1 detection primers:

Forward: 5’- AGTAGCTGGGATTACAGGGCTGCAT-3’

Reverse: 5’- GCTGGCAGATCACCTGAGGTCAGG-3’

Binding site 2 detection primers:

Forward: 5’- ATGCTGTAGCTGTTTCTTCAG-3’

Reverse: 5’- TAGGTCGATCACTTGAGCCCAGGAGT-3’

**Biotin-labeled DNA probes from the UBE2S promoters or mutant probes**

Site1 (WT): ACAGGGCTGCATCAACACACCTGGCTAATTTTTGTATTTTTAGT

ACAGATGGGGTTTCAC

Site1 (MUT): ACAGGGCTGCATCAACACACCTGGCTAAACACAATTTTTAGT

ACAGATGGGGTTTCAC

Site2 (WT): GTAGCTGTTTCTTCAGTTGTTTTGGGTTTGTTTGTTTTGTACATA

TTGGTGGGGGTCTCT

Site2 (MUT): GTAGCTGTTTCTTCAGTTGTTTTGGGTTACACACATTTGTACA

TATTGGTGGGGGTCTCT
