# Supplementary material for: UBE2S promotes cell chemoresistance through PTEN-AKT signaling in hepatocellular carcinoma
Source: Cell Death Discov. 2021 Nov 16;7:357. doi: 10.1038/s41420-021-00750-3 (PMC8595659; doi:10.1038/s41420-021-00750-3)
Supplement: Supplementary file 5 — declaration [file 41420_2021_750_MOESM5_ESM.pdf]

DECLARATION OF CONTRIBUTIONS TO ARTICLE

ADMC

Manuscript Number: CDDISCOVERY-21-2091R

Journal Name: *Cell Death & Discovery* (the 'Journal')

Proposed Title of the Contribution: UBE2S promotes cell chemoresistance through PTEN-AKT signaling in hepatocellular carcinoma (the 'Contribution)

Author(s): Liang Gui, Sicai Zhang, Yongzi Xu, Hongwei Zhang, Ying Zhu, Lianbao Kong, (the 'Authors')

For all *CDDiscovery* articles, each person named as an author in the published version must be able to show he or she has contributed substantially to the article.

Authorship credit should be based on 1) substantial contributions to conception and design, acquisition of data, or analysis and interpretation of data; 2) drafting the article or revising it critically for important intellectual content; and 3) final approval of the version to be published. Authors should meet conditions 1, 2 and 3.

Any person who cannot be shown to have made a substantial contribution to the article cannot be listed as an author in the final version. The name of any person who is deemed to have made a minor contribution can, however, appear in the Acknowledgments section of the article.

Please complete the table below to indicate the contributions of all named authors to the manuscript.

Author Full Name:	Specification of Contribution to the Manuscript:
Liang Gui	performed the experiments.
Sicai Zhang	analyzed the data and drafted the manuscript
Yongzi Xu	performed the experiments.
Hongwei Zhang	analyzed the data
Ying Zhu	designed the study and reviewed the manuscript.
Lianbao Kong	designed the study and reviewed the manuscript

Please complete the table below to indicate the contributions of all named authors to the figures.

Figure 1:

Liang Gui, Yongzi Xu. Hongwei Zhang, Sicai Zhang, Ying Zhu, Lianbao Kong

Figure 2:

Liang Gui, Yongzi Xu

Figure 3:

Liang Gui, Yongzi Xu, Hongwei Zhang, Sicai Zhang

Figure 4:

Liang Gui, Yongzi Xu. Hongwei Zhang, Sicai Zhang, Ying Zhu, Lranbao Kong

Figure 5:

Liang Gui, Yongzi Xu

Figure 6:

Liang Gui, Yongzi Xu

Signed for and on behalf of the Author(s):	Print Name:	Date:
[illegible] Liang Gui	Liang Gui	2021-10-10
[illegible] Sicai Zhang	Sicai Zhang	2021-10-10
[illegible] Yongzi Xu	Yongzi Xu	2021-10-10
[illegible] Hongwei Zhang.	Hongwei Zhang	2021-10-10
[illegible] Ying Zhu	Ying Zhu	2021-10-10
[illegible] Lianbao Kong	Lianbao Kong	2021-10-10
